# Targeting IDH1/2 mutant cancers with combinations of ATR and PARP inhibitors

**DOI:** 10.1093/narcan/zcab018

**Published:** 2021-05-17

**Authors:** Amrita Sule, Jinny Van Doorn, Ranjini K Sundaram, Sachita Ganesa, Juan C Vasquez, Ranjit S Bindra

**Affiliations:** Department of Therapeutic Radiology, Yale University School of Medicine, New Haven, CT 06511, USA; Department of Therapeutic Radiology, Yale University School of Medicine, New Haven, CT 06511, USA; Department of Therapeutic Radiology, Yale University School of Medicine, New Haven, CT 06511, USA; Department of Therapeutic Radiology, Yale University School of Medicine, New Haven, CT 06511, USA; Department of Pediatrics, Yale School of Medicine, New Haven, CT 06511, USA; Department of Therapeutic Radiology, Yale University School of Medicine, New Haven, CT 06511, USA

## Abstract

Mutations in the isocitrate dehydrogenase-1 and -2 (IDH1/2) genes were first identified in glioma and acute myeloid leukemia (AML), and subsequently found in multiple other tumor types. These neomorphic mutations convert the normal product of enzyme, α-ketoglutarate (αKG), to the oncometabolite 2-hydroxyglutarate (2HG). Our group recently demonstrated that 2HG suppresses the high-fidelity homologous recombination (HR) DNA repair pathway, resulting in a state referred to as ‘BRCAness’, which confers exquisite sensitivity to poly(ADP-ribose) polymerase (PARP) inhibitors. In this study, we sought to elucidate sensitivity of IDH1/2-mutant cells to DNA damage response (DDR) inhibitors and, whether combination therapies could enhance described synthetic lethal interactions. Here, we report that ATR (ataxia telangiectasia and Rad3-related protein kinase) inhibitors are active against IDH1/2-mutant cells, and that this activity is further potentiated in combination with PARP inhibitors. We demonstrate this interaction across multiple cell line models with engineered and endogenous IDH1/2 mutations, with robust anti-tumor activity *in vitro* and *in vivo*. Mechanistically, we found ATR and PARP inhibitor treatment induces premature mitotic entry, which is significantly elevated in the setting of IDH1/2-mutations. These data highlight the potential efficacy of targeting HR defects in IDH1/2-mutant cancers and support the development of this combination in future clinical trials.

## Graphical Abstract

**Graphical Abstract:**
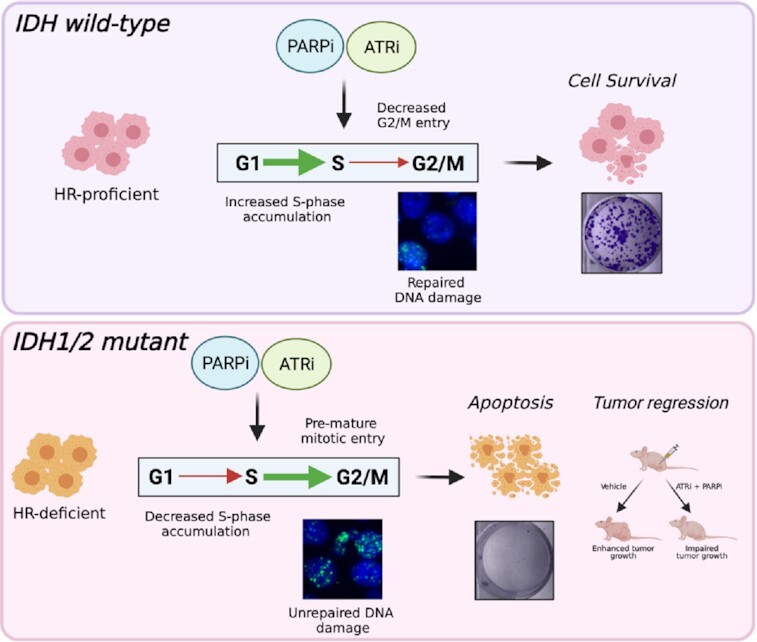
In the presence of elevated genomic instability in HR-deficient IDH1/2 mutant cells and accumulation of PARP-inhibitor mediated DNA breaks, ATR inhibition abrogates S-phase arrest and causes apoptosis by premature mitotic entry (Created using Biorender.com).

## INTRODUCTION

Isocitrate dehydrogenase-1 and -2 (IDH1/2) are enzymes which convert the Kreb's cycle intermediate isocitrate to α-ketoglutarate (αKG). IDH1/2 mutations occur frequently in subsets of human malignancies derived from a wide range of tissues (1). IDH1 mutations have been recurrently reported in low-grade glioma, glioblastoma multiforme (1), acute myeloid leukemia (AML) (2), cholangiocarcinoma (3), chondrosarcoma, and also melanoma (4,5). Mutations in the IDH2 gene also have been reported in gliomas and AML (1,6). They are heterozygous missense mutations which primarily occur at the R132 residue in IDH1, and the R140 or R172 residues in IDH2 genes (1). These mutations in IDH1/2 are neomorphic, in that they convert the normal product of the WT enzyme, αKG, into 2-hydroxyglutarate (2HG). 2HG is an oncometabolite which is thought to drive transformation and tumor progression by altering a diverse range of cellular processes, including metabolism and epigenetic changes (7–9).

Previous work from our lab and others have demonstrated that IDH1/2-mutant cancers are defective in homologous recombination (HR), which confer sensitivity to poly (ADP-ribose) polymerase (PARP) inhibitors (10–13). Mechanistically, we have shown that 2HG production results in aberrant hypermethylation of histone 3 lysine 9 (H3K9) at DNA break sites, masking a H3K9 trimethylation signal required for recruitment of homology-dependent repair factors. This results in suppression of HR and subsequent PARP inhibitor sensitivity, which is commonly referred to as a BRCAness phenotype (10,14). We also have extended these findings to other tumor-associated oncometabolites, such as succinate and fumarate (15).

PARP is involved in base excision repair (BER) and is important for repairing single-strand breaks (SSBs) (16). Cells with HR defects are thought to accumulate SSBs resulting from replication fork stalling or collapse when treated with PARP inhibitors, which leads to lethal double strand breaks (DSBs) (17,18). PARP inhibitors have been tested extensively in HR-deficient cancer models, such as those with BRCA1 and BRCA2 mutations (19), as single agents or in combination with a range of DNA damaging agents and DNA repair inhibitors (20,21). ATR (ataxia telangiectasia and Rad3-related protein kinase) is a key cell cycle regulator, which halts the cell cycle in an event of DNA damage and initiates DNA damage response (DDR). ATR maintains genomic stability by responding to replication stress and DNA damage in S-phase by activating the S-phase checkpoint, thereby allowing DNA repair and preventing premature mitotic entry (22). ATR inhibition allows damaged cells to proceed past the S-phase checkpoint, promoting the induction of DSBs and premature mitotic entry; ultimately leading to apoptosis (23,24). Independent of replication stress ATR also regulates the G2/M check point and hence abrogation of ATR could potentially lead to pre-mitotic entry (25–27). Several preclinical studies have demonstrated the efficacy of PARP inhibitors in combination with ATR inhibitors in many BRCA-deficient, as well as ATM-deficient, cancer models (25–27).

In this study, we sought to further elucidate the spectrum of DDR inhibitor sensitivity in IDH1/2-mutant cells, and whether combination therapies could further enhance this synthetic lethal interaction. Here, we report that ATR inhibitors are active against IDH1/2-mutant cells and that this activity can be further potentiated by combination with PARP inhibitors both *in vitro* and *in vivo*.

## MATERIALS AND METHODS

### Cell culture

The U87 IDH1 R132H/+ (ATCC^®^ HTB-14IG™), HT1080 IDH1 R132C (ATCC® CCL-121™) and SW1353 IDH2 R172S (ATCC^®^ HTB-94) cell lines was purchased from ATCC. HCT116 IDH1 R132H/+ was purchased from (Horizon Discovery HD 104-013). LN229 cells with doxycyclin inducible IDH1 R132H/+ have been described previously (10). LN229, HT1080 and SW1353 cells were cultured in DMEM with 10% FBS (Gibco). U87 cells were cultured in DMEM-F12 with 10% FBS (Gibco). HCT116 (parental and IDH1 R132H/+) cells were cultured in McCoy's 5A with 10% FBS (Gibco). SW1353 cells were cultured in RPMI 1640 with 10% FBS (Gibco).

### Antibodies and reagents

Anti-Histone H3 phospho-Ser-10 (ab14955, Abcam), anti-GAPDH (60004-1-Ig, Proteintech), anti-Caspase-3 (9662, Cell Signaling Technology), anti-Cleaved-Caspase-3(9664, Cell Signaling Technology), anti-PARP1 (VMA00016, BioRad), anti-Cleaved -PARP (5625, Cell Signaling Technology), anti-Rabbit-IgG, HRP conjugate (SA00001-2, Proteintech), anti-Mouse-IgG, HRP conjugate (SA00001-1, Proteintech), anti-Cyclin A (SC-271682, Santa Cruz Biotechnology), anti-RPA70 (ab12320,Abcam), γ-H2AX(05–636, Millipore Sigma), Alexa Fluor 647 (A21236, ThermoFisher Scientific), Alexa Flour (A32744, ThermoFisher Scientific), Click-iT™ EdU (C10419,ThermoFisher Scientific), RNase/PI buffer (550825, BD Biosciences), Vectashield DAPI (H-1200-10, Vector Laboratories) were used. (2*R*)-Octyl-α-hydroxyglutarate (2-HG) was purchased from Cayman chemical. Drugs were purchased from Selleckchem. AZD6738 and Olaparib were provided by AstraZeneca.

### Short-term cell viability assays

Cells were plated in 96-well plates at a concentration of 2000 cells per well and allowed to adhere at room temperature for 60 min before returning them to the incubator. After 24 h, the media was changed and replaced with respective drugs in triplicates at indicated concentrations. Five days after drug treatment, the cells were fixed with 3.7% paraformaldehyde and stained with Hoechst (1 μg/ml). The plates were then imaged on a Cytation 3 automated imager (BioTek), and cells were counted using CellProfiler (http://cellprofiler.org/).

### Clonogenic survival assays

Cells were counted and diluted in media containing various concentrations of respective drugs. They were immediately transferred into six-well plates in triplicate at three-fold dilutions ranging from 9000 to 37 cells per well. After 12–14 days, plates were washed with PBS and stained with crystal violet. Colonies were counted by hand. Counts were normalized to plating efficiency of corresponding treatment condition, unless otherwise noted.

### U2OS DR-GFP assay

U2OS DR-GFP assay was carried out as previously described (28). To test the effect of 2HG, U2OS cells expressing the reporter were cultured with or without 300 μM 2HG. Drugs were added 24 h prior to ligand addition. Ligands were washed off after 24 h and media was replenished with respective drugs and 2HG. Cells were analyzed on a BD FACS Calibur flow cytometer after 72 h.

### Flow cytometry

For propidium iodide (PI) staining, cells were seeded in 60 mm dishes. 24–48 h after plating, cells were treated with drugs. Twenty four hours post drug treatment, cells were harvested by trypsinization and fixed with 70% ethanol. Cells were then stained in RNase/PI buffer (BD Biosciences). For EdU staining, cells were treated with 10 μM EdU 1.5 h prior to harvesting and processed according to manufacture's protocol (Thermo Fisher:Click iT™ EdU Cell Proliferation Kit). For p-H3 staining, cells were trypsinized 24 h post drug treatment and processed as previously described by Forment *et.al*. (29). Cells were treated with an extraction buffer, 0.2% Triton (PBS) for 10 min, washed with PBS (1% BSA) followed by fixation with 70% ethanol. Cells were incubated in primary and secondary antibody solutions for 1 h and 30 min, respectively, at room temperature. Cells were run on a BD FACS Calibur flow cytometer and analyzed with FlowJo software.

### NAD quantification

NAD level analyses were performed using a NAD/NADH Quantification kit (Sigma), as per manufacturer's specifications.

### Immunofluorescence

25,000 cells were seeded on glass chamber slides. Slides were treated with drugs for 24 h. Cells were fixed with 3.7% paraformaldehyde. Cells were then washed with PBS and permeabilized with 0.2% Triton–PBS. Cells were incubated with primary antibody (anti-γ-H2AX, anti-pH3 or anti-cyclin A) overnight at 4°C and secondary antibody (Alexa Flour 594 or Alexa Flour488) at room temperature for 1 h. For RPA70 immunostaining, cells were pre-extracted for 1 min in 0.1 M PIPES pH 6.9, 1 mM EGTA, 4 M glycerol and 0.2% Triton X-100. Cells were washed once for 2 min in 0.1 M PIPES pH 6.9, 1 mM EGTA, and 4 M glycerol in H2O, and then fixed for 20 min with 3.7% formaldehyde, 50 mM PIPES pH 6.9, 5 mM EGTA, and 1 mM MgCl_2_. Cells were blocked in casein + 5% goat serum before adding primary antibody (anti-RPA70) at 1:500 overnight at 4°C. The next day, cells were washed with PBS before secondary antibody (Alexa-flour-488) incubation at 1:500 for 2 h at room temperature. Cells were analyzed on a Keyence BZ-X800. Foci were analyzed with the Focinator v2-21 software as previously described (30).

### Protein detection by western blot

Whole cell lysates were prepared using RIPA buffer (Cell Signaling Technology) with 1× protease and phosphatase inhibitor (78442, ThermoFisher Scientfic). Proteins were separated by SDS-PAGE and transferred to a PVDF membrane for analysis. Blots were blocked in 5% BSA (Gold Biotechnology) in 1× TBST (American Bio) and probed overnight with respective primary antibodies. Blots were washed with 1× TBST and incubated with HRP conjugated secondary antibodies for 1 h at room temperature. Blots were exposed using Clarity Western ECL substrate (BioRad), and imaged on a ChemiDoc (BioRad) imaging system.

### *In vivo* Olaparib and AZD6738 efficacy studies

Female athymic nu/nu mice (Hsd:Athymic Nude-Foxn1nu, Envigo) were used for *in vivo* xenograft studies. One million HCT116 IDH1 R132H/+ or HT1080 IDH1 R132C/+ cells were injected subcutaneously into the flank at a concentration of 1 × 10^6^ cells/100 μl of PBS. Prior to drug treatment, mice were randomized and placed into groups of 5–8 animals such that the average starting tumor volume of each group was approximately equal (100 mm^3^). Olaparib and AZD6738 were administered daily up to 21 or 28 days. Each dose of olaparib was delivered via oral gavage at 25 or 50 mg/kg. Each dose of AZD6738 was delivered via oral gavage at 25 or 50 mg/kg. Olaparib and AZD6738 were solubilized in DMSO and diluted with 10% (w/v) 2-hydroxy-propyl-b-cyclodextrin (Sigma) to obtain the desired concentration. Tumors were measured by calipers and volume was calculated using the equation for ellipsoid volume: volume = π/6 × (length) × (width)^2^. IACUC protocols at the Yale School of Medicine were followed throughout the study.

### Statistical analysis

Data are presented as mean ± SD or SEM. Comparisons were made using Student's *t* test (Clonogenic survival assay). Two-way analysis of variance (ANOVA) was used to test for significant differences between groups (xenograft studies). All tests were two-sided. Statistical analyses were carried out using GraphPad Prism. P values are indicated either directly on figures or using *P < 0.05, **P < 0.01, ***P < 0.001 and ****P < 0.0001.

## RESULTS

### IDH1 mutation confers sensitivity to ATR inhibitors

**Figure 1.**
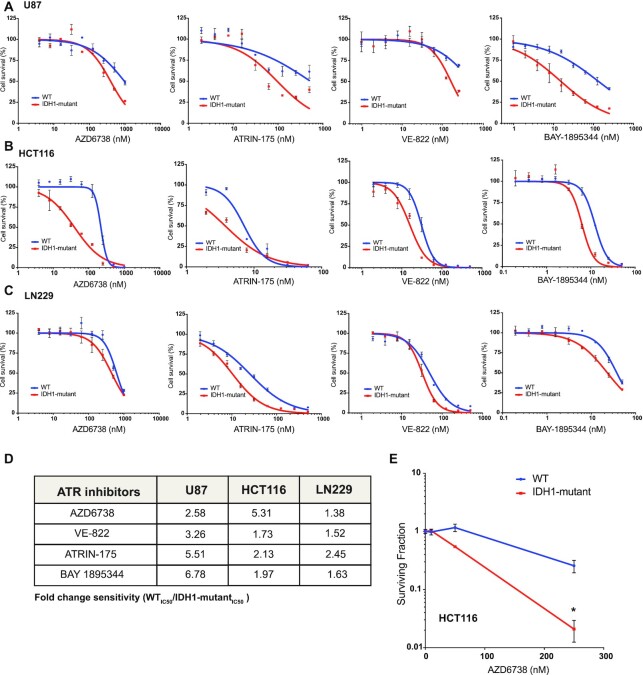
IDH1 mutation confers sensitivity to ATR inhibitors. (**A–C**) Five-day short-term cell viability assays of four separate ATR inhibitors AZD6738, ATRIN175, VE-822 and BAY-1895344. U87 (WT and IDH1 R132H/+), HCT116 (WT and IDH1 R132H/+) and LN229 (WT and doxycycline inducible IDH1 R132H/+) cells were used for this assay. (**D**) IC^50^ ratios of screened WT and the IDH1 mutant cells indicate that the IDH1 mutant cells are more sensitive to all the four ATR inhibitors tested. (**E**) HCT116 WT and R132H/+ cells were treated with AZD6738 for 14 days (*n* = 6). Error bars represent means ± SEM, *P < 0.05.

Previous studies from our group and others have established mutant IDH1/2-induced HR defects and PARP inhibitor sensitivity (10–13), although the spectrum of sensitivity to other DDR inhibitors has yet to be fully elucidated. ATR inhibitors have been shown to be synthetic lethal with DNA repair defects caused by ATM, TP53 or Rad51 loss in cancer cells (31–34), which prompted us to test whether they would be active against IDH1/2-mutant cells. We tested four different ATR inhibitors; AZD6738, ATRIN-175, VE-822 and BAY-1895344 in U87, HCT116 and LN229 IDH1 R132H/+ isogenic cell line models using short-term viability assays *in vitro*. We observed evidence of ATR inhibitor sensitivity which was greatest in IDH1-mutant versus -WT cells (Figure 1A–C), with WT/mutant IC50 ratios ranging from 1.28 to 6.78 (Figure 1D). We confirmed this differential sensitivity between IDH1-mutant and -WT cells in a clonogenic survival assay, which revealed a >10-fold sensitivity of IDH1-mutant cells to AZD6738 at a dose of 250 nM (Figure 1E). These data confirm that like PARP inhibition, ATR inhibition is synthetic lethal with mutant IDH1/2-associated HR defects.

### ATR and PARP inhibition are synthetically lethal in IDH1/2 mutant cancers

**Figure 2.**
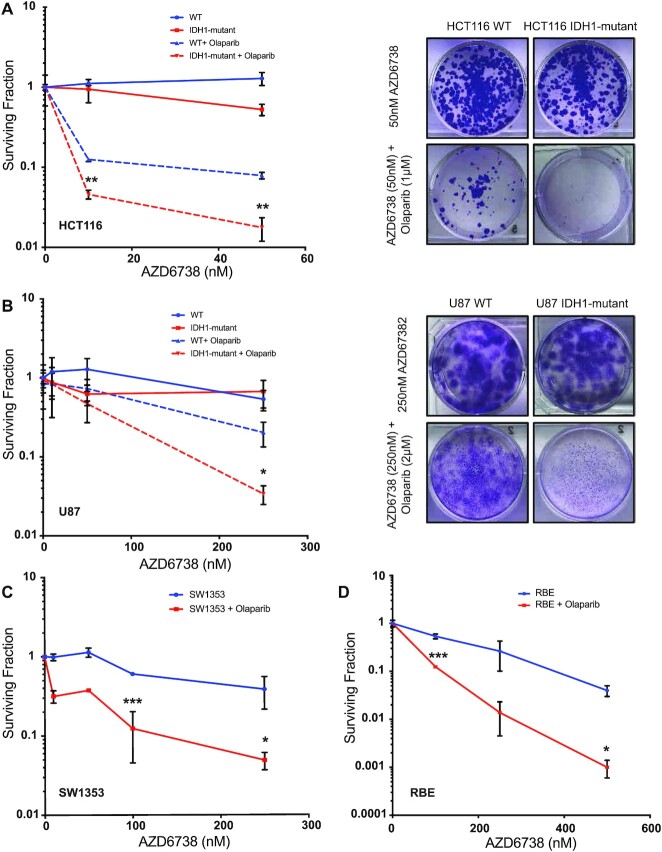
ATR inhibitor increases PARP inhibitor mediated sensitivity in IDH1 mutant cells. (**A**) Quantification and representative images of clonogenic survival assays of HCT116 WT and IDH1-mutant (R132H/+) cells were treated with AZD6738 alone (solid lines) and in combination with 1 μM olaparib (dashed lines) for 14 days (*n* = 6). (**B**) Quantification representative images of clonogenic survival assays of U87 WT and IDH1-mutant (R132H/+) cells were treated with AZD6738 alone (solid lines) and in combination with 2 μM olaparib (dashed lines) for 14 days (*n* = 4). (**C**) Quantification of clonogenic survival assay of SW1353 (IDH2 R172S/+) cells treated with AZD6738 alone (blue line) and in combination with 1 μM olaparib (red line) for 14 days. (**D**) Quantification of clonogenic survival assay of RBE (IDH1 R132S/+) cells treated with AZD6738 alone (blue line) and in combination with 2 μM Olaparib (red line) for 14 days. Error bars represent means ± SEM. ***P < 0.001, **P < 0.01, *P < 0.05.

A number of studies have shown that ATR and PARP inhibitor combinations are more cytotoxic than PARP inhibitor monotherapy in BRCA1/2-deficient cells (26,35). Given our previous findings of oncometabolite-induced PARP inhibitor sensitivity, and our new findings above which demonstrate robust ATR inhibitor sensitivity, we hypothesized that this combination would also be effective against IDH1/2-mutant cells. As shown in Figure 2A, combination therapy of a PARPi, olaparib and an ATRi, AZD6738 in HCT116 WT and IDH1-mutant cells was significantly more cytotoxic in IDH1-mutant cells in clonogenic survival assays (Figure 2A), which was also observed in the U87 WT and IDH1-mutant cell line pair (Figure 2B). We validated the ATRi and PARPi combination efficacy with another ATR inhibitor, BAY1895344, in combination with olaparib in HCT116 isogenic cell line (Supplementary Figure S2A).

We also sought to extend these findings to cell line models with endogenous IDH1/2 mutations. To this end, we selected several IDH1/2-WT and -mutant cholangiocarcinoma, fibrosarcoma and chondrosarcoma cell lines, and we first tested monotherapy PARP and ATR inhibitor sensitivity in order to identify the appropriate dose ranges for the combination drug studies (Supplementary Figure S1A and B). We then selected the chondrosarcoma cell line SW1353, which harbors an IDH2 mutation (R172S/+), as well as the cholangiocarcinoma cell line RBE, which harbors an IDH1 mutation (R132S/+) for combination ATR and PARP inhibitor studies. Both cell lines exhibited increased sensitivity to the olaparib and AZD6738 combination relative to AZD6738 monotherapy in clonogenic survival assays (Figure 2C, D, Supplementary Figure S2B, C and S3E). Of note, olaparib monotherapy was not as effective in short-term cell viability assays with RBE cells in comparison to the other cell lines tested (Supplementary Figure S1A). However, clonogenic survival assays in combination with AZD6738 revealed a thousand-fold sensitization in RBE cells to olaparib, indicating the exquisite potentiation associated with the combination therapy compare to single agent treatment (Figure 2D). As reported previously (36,37), we also saw some cytotoxicity of the combination in the WT cells, however the cytotoxicity in the IDH1-mutant cells was more heightened.

We also tested the efficacy of two other PARP inhibitors, niraparib and talazoparib, by themselves and in combination with AZD6738 in HCT116 WT and IDH1-mutant cell line (Supplementary Figure S3A–E). Additionally, we also assessed the NAD levels in the isogenic cell line pairs to validate if PARP inhibitor sensitivity was affected by the NAD. We did not observe any significant difference in the NAD levels of HCT116 and U87 (WT and IDH1-mutant) cells (Supplementary Figure S3F). Taken together, our data suggests that ATR and PARP inhibitor combinations are highly effective in IDH1/2-mutant cells *in vitro*.

### ATR and PARP inhibitor combination increase DSBs and suppress HR

**Figure 3.**
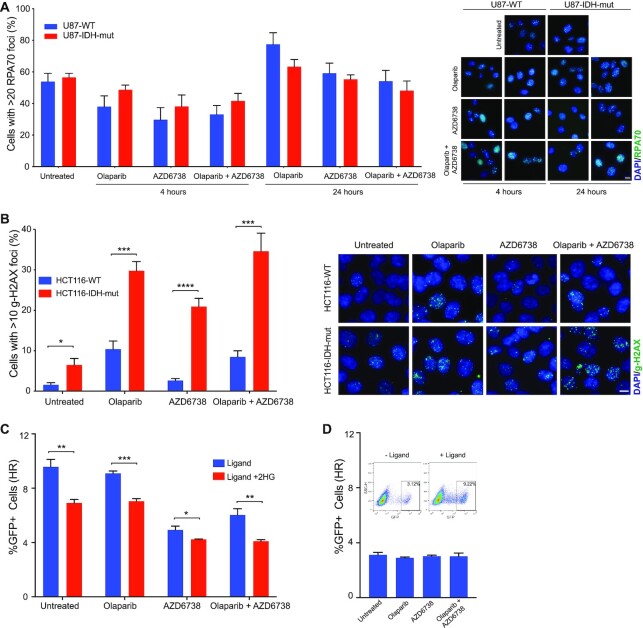
ATR and PARP inhibition increases unrepaired damage and suppresses HR. (**A**) U87 WT and IDH1-R132H/+ cells were treated with olaparib (10 μM), AZD6738 (5 nM) or both for 4 and 24 h. Cell were fixed and stained for RPA70 and counterstained with DAPI. Cells with more than 20 RPA70 foci were counted in seven distinct fields. In total 250–350 cells were analyzed. Representative images of cells stained with RPA (green) and counterstained with DAPI (Blue) are shown. The images shown were acquired using a 40× objective lens. The scale bar is 10 μm. (**B**) HCT116 WT and IDH1-R132H/+ cells were treated with olaparib (1 μM), AZD6738 (500 nM) or both for 24 h. Cell were fixed and stained for γ-H2AX and counterstained with DAPI. Cells with more than 10 γ-H2AX foci were counted in eight distinct fields. In total 800–1000 cells were analyzed. Representative images of cells stained with γ-H2AX (green) and counterstained with DAPI (Blue) are shown. The images shown were acquired using a 40× objective lens. The scale bar is 10 μm. (**C**) Quantitation of ligand inducible U2OS-DR-GFP assay where cells were cultured with or without 300 μM 2HG for 3 days and with and without olaparib (1 μM), AZD6738 (500 nM) or both for 24 h prior to ligand induction (*n* = 3). (**D**) Quantitation of control U2OS-DR-GFP assay without ligand induction. Dotplots show GFP population with and without ligand addition. Error bars represent means ± SEM. ***P < 0.001, **P < 0.01, *P < 0.05.

ATR protects replicating DNA and in an advent of DNA damage, delays cell cycle progression via replication fork stabilization (38). Increased replication stress in cancers makes them sensitive to ATR inhibitors (39). To assess if IDH1/2-mutant cells are sensitive to ATR inhibitors due to replication stress, we looked at RPA70 foci formation in U87 WT and IDH-mutant cells treated with olaparib, AZD6738 or both (Figure 3A). We did not observe any significant differences between the WT and IDH1-mutant cells in response to olaparib or the combination treatment after 4 hours of treatment (Figure 3A). After 24 h of treatment the RPA70 foci increased overall in all treatment groups however, we did not observe any significant difference between WT and IDH1-mutant cells. ATR inhibitor exerts sensitivity possibly by a mechanism independent of replication stress. We then assessed presence DNA damage by looking at γ-H2AX foci in HCT116 WT and IDH1-mutant cells post olaparib, AZD6738 and combination treatment. After 24 h, IDH1-mutant cells had significantly increased proportion of cells with γ-H2AX foci relative to WT cells suggesting increased levels of unrepaired DNA damage after drug treatment (Figure 3B).

We further looked at effect of olaparib, AZD6738 and the combination treatment on homologous recombination (HR) repair efficacy. We used a ligand-dependent I-SceI system combined with the U2OS DR-GFP assay to measure HR (40) (Figure 3C). As these cells do not have IDH1/2 mutations, we added 2HG directly to the cells. As previously reported (10), we observed that 2HG alone suppresses HR. Cells not treated with 2HG have 9.5% GFP+ cell population which goes down to around 6.5% when treated with 2HG. We observed that in cells treated with 2HG; olaparib (6.7%), AZD6738 (4.1%) as well as the combination treatment (3.9%) significantly reduced the number of GFP+ cells thereby significantly suppressing HR (Figure 3C and D). AZD6738 treatment also caused a suppression of HR in cells not treated with 2HG. These data, coupled with findings in Figure 3B that γ-H2AX levels are highest in the PARP/ATR inhibitor-treated IDH1-mutant cells, suggest that sensitization occurs via a combination of decreased HR and unrepaired DSBs.

### ATR and PARP inhibitor treatment cause premature mitotic entry

**Figure 4.**
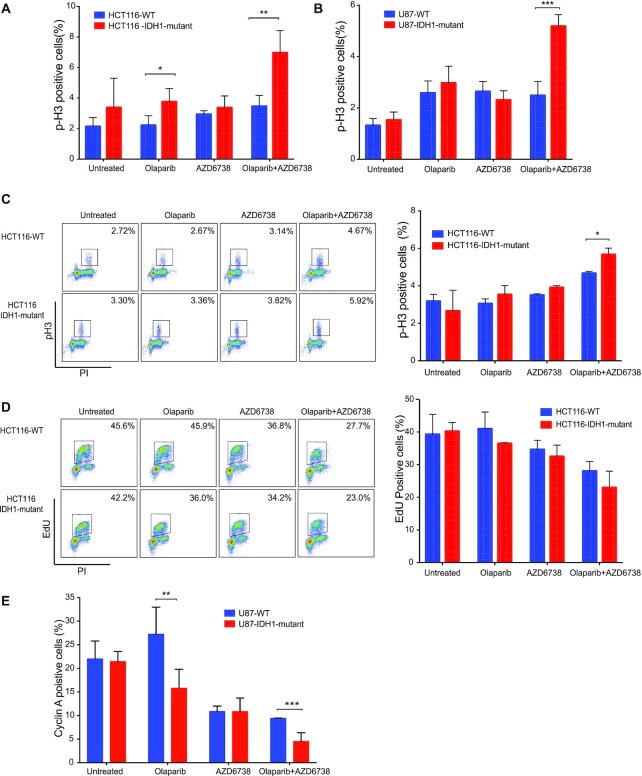
ATR and PARP inhibition cause IDH1/2 mutants to prematurely enter mitosis. (**A** and **B**) HCT116 and U87 (WT and IDH1-R132H/+) cells were treated with olaparib (1μM), AZD6738 (500 nM) or both for 24 h. Cell were fixed and stained for p-H3 (Ser10) and counterstained with DAPI. Number of p-H3 positive cells were analyzed in 5 distinct fields (*n* = 2). (**C**) Representative p-H3-Alexa-647 plots of HCT116 WT and R132H/+ cells that were treated with olaparib (1 μM), AZD6738 (500 nM) or both for 24 h. DNA content (propidium iodide) and pH3-Ser10/Alexa-488 were assessed by flow cytometry (*n* = 3). Quantification of result shown on the right panel. (**D**) Representative EdU-Alexa-647 plots of HCT116 WT and R132H/+ cells were treated with olaparib (1 μM), AZD6738 (500 nM) or both for 24 h DNA content (propidium iodide) and EdU -Ser10/Alexa-488 were assessed by flow cytometry (*n* = 3). (**E**) HCT116 (WT and IDH1-R132H/+) cells were treated with olaparib (1 μM), AZD6738 (500 nM) or both for 24 h. Cell were fixed and stained for cyclin-A and counterstained with DAPI. Number of cylin-A positive cells were analyzed in five distinct fields (*n* = 3). Quantification of result shown on the right panel. Error bars represent means ± SEM. ***P < 0.001, **P < 0.01, *P < 0.05.

Next, we sought to further probe the mechanistic basis for enhanced tumor cell kill induced by AZD6738 and olaparib combination treatment in IDH1/2-mutant cells. We first examined the effect of AZD6738 and Olaparib on cell cycle distribution in our IDH1-mutant and -WT isogenic cell line pairs. We found that treatment of HCT116 WT with 500 nM AZD6738 and 1 μM Olaparib, either alone or in combination, did not induce significant changes in cell cycle profiles after 24 h (Supplementary Figure S4A and B). However, both olaparib and AZD6738 induced increases in the G2/M phase fractions of HCT116- IDH1-mutant cells, which was further augmented with the combination treatment (Supplementary Figure S4A and B). We therefore assessed the mitotic cell population by immunofluorescence (IF) staining for p-Histone 3 (p-H3) at Ser 10, which identifies cells in late G2 and mitosis. These experiments revealed a significant increase in p-H3 positive cells after treatment with olaparib and AZD6738 in HCT116 IDH1-mutant versus -WT cells (Figure 4A), which was also observed in the U87 isogenic pair (Figure 4B). We also observed a small, but significant, increase in p-H3 levels after olaparib treatment alone in HCT116 IDH1-mutant versus -WT cells. Representative images IF images for these experiments are shown in Supplementary Figure S5. We then validated this result using multiparametric flow cytometry to measure p-H3 levels and cell cycle phase, which again revealed a significant increase in p-H3 levels after treatment with olaparib and AZD6738 in HCT116 IDH1-mutant versus -WT cells (Figure 4C).

We went on to profile DNA synthesis using EdU proliferation assays after treatment with olaparib and AZD6738, either alone or in combination, in order to assess whether there also were mutant IDH1-dependent differences in replication. We saw that olaparib and AZD6738 alone reduced the number of cells replicating in S-phase in IDH1-mutant cells relative to untreated control, which was further reduced with the combination (Figure 4D). Although not significant, the number of cells in S-phase were less in the IDH1-mutant cells relative to IDH-WT cells. We then validated this result by IF staining for cyclin A which identifies cells primarily in S phase and early G2-phase (41,42). We observed that olaparib monotherapy increased cyclin A positive cells in the IDH1 WT but not in the IDH1-mutant cells. In line with our previous EdU staining experiment we saw that Olaparib and AZD6738 combination significantly reduced the number of replicating cells in the IDH1-mutant cell line (Figure 4E). Representative images IF images for these experiments are shown in Supplementary Figure S6. We also saw significantly differences in p-H3 and Cyclin A population in the WT in single vs combination treatment.

**Figure 5.**
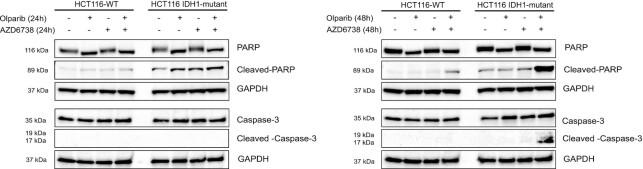
ATRi and PARPi combination caused increased apoptosis in IDH1-mutant cells. HCT116 (WT and IDH1-R132H/+) cells were treated with olaparib (1 μM), AZD6738 (500 nM) or both for 24 h (left panel) or 48 h (right panel). Lysates were harvested after 24 and 48 h. Cleaved caspase-3 and cleaved PARP was evaluated by western blot analysis.

Finally, we assessed the expression of apoptotic markers, cleaved-PARP and cleaved-caspase-3 in response to the drug treatments. HCT116 WT and IDH1 mutant cells treated with olaparib, AZD6738 or AZD6738 in combination with olaparib for 24 and 48 h. Cell lysates were analyzed by western blotting. The combination of olaparib and AZD6738 did not cause any increase in the apoptotic proteins at 24 h (Figure 5, left panel). However, post 48 h of treatment there was significantly high expression of cleaved-PARP and cleaved caspase-3 in the IDH1 mutant cells treated with AZD6738 and olaparib combination relative to single drug treatments and the WT cells. (Figure 5, right panel). Taken together, these data suggest that olaparib and AZD6738 specifically induce a defect in mitotic entry, most pronounced in IDH1-mutant cells, which drives them to apoptosis.

### ATRi and PARPi is active against IDH1-mutant tumors *in vivo*

**Figure 6.**
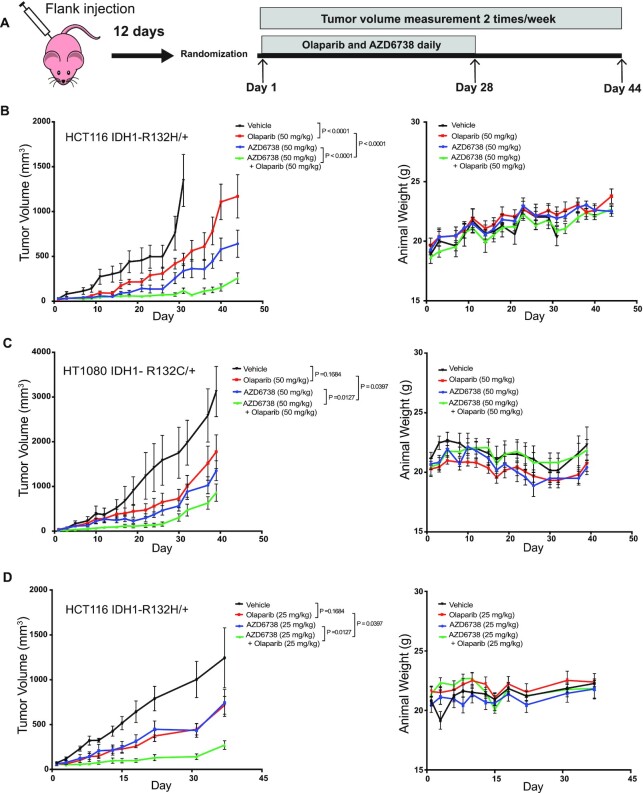
Combination of ATRi and PRAPi was effective in IDH1-mutant mouse xenograft model. (**A**) Athymic nude mice received subcutaneous injection of HCT116 IDH1 R132H/+ or HT1080 IDH1 R132C/+ cells. Twelve days after injection, the hind flank tumors were measured and equally distributed to four-arm treatment groups (**B**) Left panel: Mice carrying flank tumors of HCT116 R132H/+ cells were treated with no treatment (*n* = 8), Olaparib alone (50 mg/kg) (*n* = 8), AZD6738 (50 mg/kg) (*n* = 8), or Olaparib (50 mg/kg) and AZD6738 (50 mg/kg) (*n* = 8). Mice were treated daily for 28 days. Mean tumor volume per group with SEM is plotted on y-axis. Right panel: mean body weight with SEM of mice during HCT116 IDH1 R132H/+ flank tumor experiment. (**C**) Left panel: mice carrying flank tumors of HT1080 'cells were treated with no treatment (*n* = 7), Olaparib alone (50 mg/kg) (*n* = 7), AZD6738 (50 mg/kg) (*n* = 7), or Olaparib (50 mg/kg) and AZD6738 (50 mg/kg) (*n* = 8). Mice were treated daily for 28 days. Right panel: mean body weight with SEM of mice during HT1080 flank tumor experiment. (**D**) Left panel: mice carrying flank tumors of HCT116 R132H/+ cells were treated with no treatment (*n* = 5), Olaparib alone (25 mg/kg) (*n* = 5), AZD6738 (25 mg/kg) (*n* = 5), or Olaparib (25 mg/kg) and AZD6738 (25 mg/kg) (*n* = 5). Mice were treated daily for 21 days. Mean tumor volume per group with SEM is plotted on y-axis. Right panel: mean body weight with SEM of mice during HCT116 R12H/+ flank tumor experiment. Error bars represent means ± SEM. *P* values were calculated using two-way ANOVA.

Finally, we tested the efficacy of the AZD6738 and olaparib in two independent flank animal models. We subcutaneously implanted HCT116 IDH1-mutant (IDH1 R132H/+) and HT1080 (IDH1 R132C/+) cells in the hind flank of athymic nude mice and treated them with vehicle, AZD6738, olaparib, or AZD6738 plus olaparib (Figure 6A). Mice (*n* = 8) were treated daily with vehicle, olaparib (50 mg/kg), AZD6738 (50 mg/kg), or olaparib (50 mg/kg) plus AZD6738 (50 mg/kg), post tumor implantation (∼100 mm^3^) for up to 44 and 40 days respectively. The combination treatment caused a significant delay in tumor growth relative to Olaparib or AZD6738 alone in the HCT116 IDH1-mutant flank model without any significant change in body weight (Figure 6B, Supplementary Figure S7A and B). Similarly, in mice bearing the fibrosarcoma HT1080 tumors, the combination of olaparib and AZD6738 significantly delayed tumor growth relative to single agent therapy but did not result in a significant drop in body weight (Figure 6C and Supplementary Figure S6C). We saw similar levels of tumor suppression in another independent study with HCT116 IDH1-mutant xenograft model, with smaller sample size (*n* = 5), where the combination therapy at lower dose of AZD6738 (25 mg/kg) and olaparib (25 mg/kg) was also effective in causing a significant tumor growth delay (Figure 6D and Supplementary Figure S8).

## DISCUSSION

This study identifies a novel synthetic lethal interaction between ATR inhibitors and IDH1/2 mutations, which can be significantly enhanced by combination with PARP inhibitors. We have seen the significant effects of olaparib and AZD6738 treatment not only in engineered, isogenic IDH1/2-mutant cell lines, but also in patient-derived cell lines with endogenous IDH1/2 mutations. In each of these models, the combination of AZD6738 and olaparib proved to be significantly cytotoxic than either drug alone. We have corroborated our *in vitro* cytotoxic studies with *in vivo* xenograft models, where we observed a significant delay in tumor growth, using both the engineered HCT116 IDH1-mutant xenograft model, as well as the fibrosarcoma HT1080 xenograft model with an endogenous IDH1 R132C mutation. While the inhibition of ATR and PARP causes moderate cytotoxicity in the WT cells, they exhibit much exacerbated cytotoxic effects in IDH1/2-mutant cells. These preclinical studies show that combined AZD6738 and olaparib treatment may have more long-lasting effects relative to monotherapy.

Previous studies have established enhanced efficacy with the combination of AZD6738 and olaparib in BRCA- mutant (25,43,44) and ATM-deficient (27) cancer models. One advantage to this combination approach is that ATR inhibitors may prevent the emergence of PARP inhibitor resistance (45) when HR-defective cells are treated with the latter class of drugs as a monotherapy (43). In this study we have reported that IDH1/2 mutant cells are sensitive to ATR inhibitors. We have shown that ATR inhibitors exert sensitivity in IDH1/2 mutant cells via suppressing HR, resulting in increased unrepaired DNA. Mechanistically, ATR inhibition has been shown to potentiate the effect of PARP inhibition via several mechanisms in BRCA-mutant cells, including the disruption of replication fork protection (44). Regarding the mechanistic basis for the enhanced efficacy of this combination IDH1/2-mutant cells, the data presented here suggest that premature mitotic entry could play a role, especially given the important role of ATR in regulating the S-G2 transition (22). Taken together we think that presence of high genomic instability in HR deficient IDH1/2 mutant cells (10) and accumulation of olaparib mediated DNA breaks, ATR inhibition can lead to abrogation of S-phase arrest and cause apoptosis by premature mitotic entry.

Achieving maximum efficacy and minimum toxicity are paramount aspects of clinical drug development. Olaparib has been extensively investigated in a multitude of trials and maximum tolerated doses have been identified (46). AZD6738 is a highly selective orally bioavailable inhibitor of ATR kinase (47) and is currently being investigated in early-phase clinical trials. A dose escalation ongoing phase I PATRIOT study (NCT02223923) is looking at safety of AZD6738 as a monotherapy and palliative radiotherapy with solid tumors (48). In another phase I study by Yap *et al.* (49) where AZD6738 toxicity was assessed in combination with other therapies, the AZD6738 and olaparib arm was found to be well tolerated (50). The AZD6738 and olaparib combination is currently being tested in phase II trials, which includes the CAPRI study for recurrent ovarian cancers (NCT03462342), as well as OLAPCO study in patients with HR defects beyond those associated with BRCA1/2 mutations (NCT02576444). Bindra and colleagues recently reported the interim results of the OLAPCO trial (NCT02576444), which tested the safety and efficacy of monotherapy olaparib against IDH1/2-mutant tumors. Efficacy was seen in four out of six IDH1/2-mutant mesenchymal sarcomas, but no clinical benefit was seen in four patients with IDH1/2-mutant cholangiocarcinomas. In addition, while the antitumor effects were robust, tumor recurrences were seen in all responding patients within 14 months. While this a preliminary readout of promising efficacy in a small number of patients, these data suggest that monotherapy PARP inhibitor treatment may be efficacious in some tumor types, but not in others, suggesting that combination strategies are needed (51). Additional efforts to test the combination of olaparib and AZD6738 is underway in phase II trial in IDH1- and IDH2-mutant cholangiocarcinoma and solid tumors, led by LoRusso and colleagues at our institution (NCT03878095). In this pre-clinical study, we demonstrate that combined treatment with AZD6738 and olaparib is well-tolerated by mice and is not associated with significant weight loss. Moreover, we demonstrate that combination treatment remains effective even at decreased dosing ranges, which could have important implications for the development of clinical trials and strategies to minimize toxicities.

## DATA AVAILIBILITY

The raw .fcs files have been submitted to FlowRepository under the following repository IDs: FR-FCM-Z3DN (EdU staining), FR-FCM-Z3DM (PI staining), FR-FCM-Z3DX (p-H3) and FR-FCM-Z3MT (U2OS-DRGFP assay).

## Supplementary Material

zcab018_Supplemental_Files
