# Supplementary material for: Targeting IDH1/2 mutant cancers with combinations of ATR and PARP inhibitors
Source: NAR Cancer. 2021 May 17;3(2):zcab018. doi: 10.1093/narcan/zcab018 (PMC8127964; doi:10.1093/narcan/zcab018)
Supplement: zcab018_Supplemental_Files [file zcab018_supplemental_files.zip › Supplemental data legend_Revised.docx]

**Supplementary data:**

**Figure S1 A.** Five-day short-term cell viability assays in IDH -WT cholangiocarcinoma (HUCCT1 and TKF1) and IDH mutant cholangiocarcinoma (RBE IDH1 R132S/+), Fibrosarcoma (HT1080 IDH1 R132C/+) and chondrosarcoma (SW1353 IDH2 R172S/+) cell with olaparib. **B**) Five-day short-term cell viability assays in RBE, HT1080 and SW1353 cells with AZD6738. IC^50^ for each drug is mentioned on the plot. Error bars represent means ± SEM.

**Figure S2. A**) Quantification and representative images of clonogenic survival assays of HCT116 WT and R132H/+ cells treated with BAY1895344 alone (solid lines) and in combination with 1 μM Olaparib (dashed lines) for 14 days (n=6)­. Representative images of SW1353 **B**) and RBE **C**) clonogenic survival assay plates treated with AZD6738, olaparib and both AZD6738 and olaparib. Error bars represent means ± SEM.****P<0.0001.

**Figure S3.** Quantification of clonogenic survival assays of HCT116 WT and IDH1-mutant (R132H/+) cells were treated with niraparib alone **A**), talazoparib alone **C**) and olaparib **E**) for 14 days (n=6) (solid lines). **B)** Quantification and representative images of clonogenic survival assays of HCT116 WT and IDH1-mutant (R132H/+) cells were treated with AZD6738 alone (solid lines) and in combination with 500 nM niraparib (dashed lines) or **D**) with AZD6738 alone (solid lines) and in combination with 0.5 nM talazoparib (dashed lines) for 14 days (n=6)­. **F**) NAD quantification in HCT116 and U76 WT and IDH1-R132H/+ cell lines (n=3). Error bars represent means ± SEM, *P<0.05, **P<0.01, ***P<0.001.

**Figure S4**. **A**) HCT116 WT and R132H/+ cells were treated with olaparib (1μM), AZD6738 (500 nM) or both for 24 hours. DNA content histograms are shown. **B**) HCT116 WT and IDH1-mutant (R132H/+) cells were treated with olaparib (1μM), AZD6738 (500 nM) or both for 24 hours. Cells in different cell cycle phases are represented (n=3).

**Figure S5.** HCT116 (**A**) and U87 (**B**) cells (WT and IDH1 R132H/+) cells were treated with olaparib (1μM), AZD6738 (500 nM) or both for 24 hours. Representative images of cells stained with p-H3 (Ser10) (Red) and counterstained with DAPI (Blue) are shown. The images shown were acquired using a 40X objective lens. The scale bar is 20 μm.

**Figure S6.** HCT116 (WT and IDH1 R132H/+) cells were treated with olaparib (1μM), AZD6738 (500 nM) or both for 24 hours. Representative images of cells stained with cyclin A (Red) and counterstained with DAPI (Blue) are shown. The images shown were acquired using a 40X objective lens. The scale bar is 20 μm.

**Figure S7. A**) Representative images of mice with HCT116 IDH1 R132H/+ xenografts on day 30 of treatment regimen. The scale in 1 cm. **B and C**) Mean tumor volume ratios for mice with HCT116 IDH1 R132H/+ and HT1080 IDH1 R132C/+ xenografts. Ratios were plotted by dividing post-treatment tumor volume by pre- treatment tumor volume. Error bars represent means ± SEM. P values were calculated using two-way ANOVA.­­

**Figure S8. A)** Athymic nude mice received subcutaneous injections of HCT116 R132H/+. Twelve days after injection, the hind flank tumors were measured and equally distributed into four treatment arms. **B)** Mean tumor volume ratios were plotted by taking ratios of tumor volume post-treatment, to the tumor volume pre- treatment. Error bars represent means ± SEM. P values were calculated using two-way ANOVA.
