# Supplementary figures and images for: Targeting IDH1/2 mutant cancers with combinations of ATR and PARP inhibitors
Source: NAR Cancer. 2021 May 17;3(2):zcab018. doi: 10.1093/narcan/zcab018 (PMC8127964; doi:10.1093/narcan/zcab018)

# Supplemental Figure 1

A

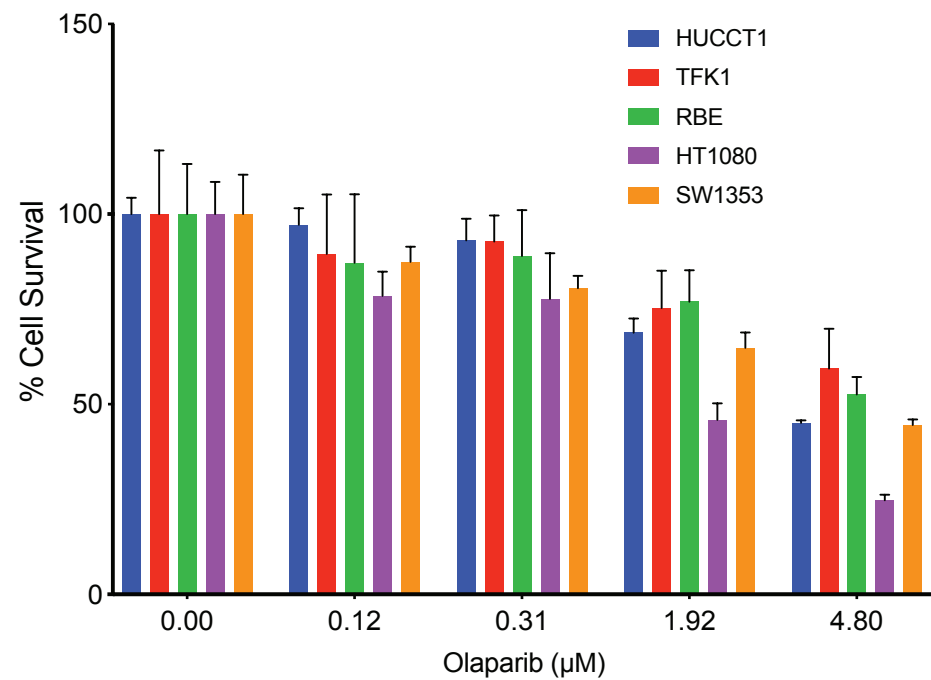

B

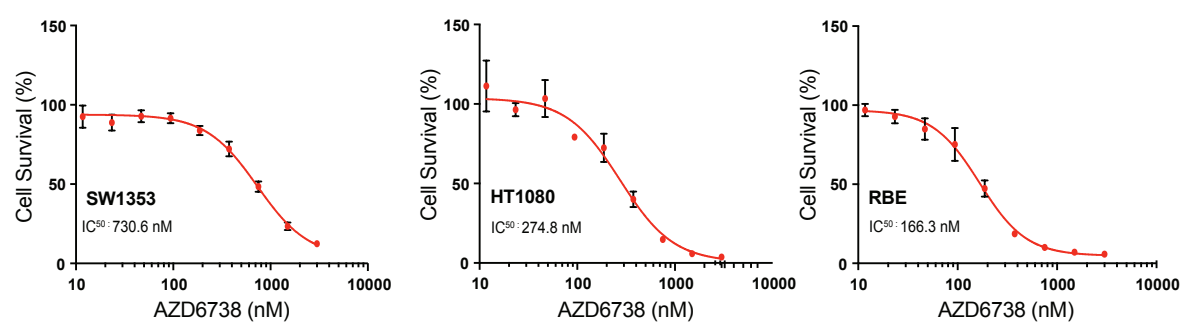

Supplement: zcab018_Supplemental_Files [file zcab018_supplemental_files.zip › S1.pdf]

# Supplemental Figure 2

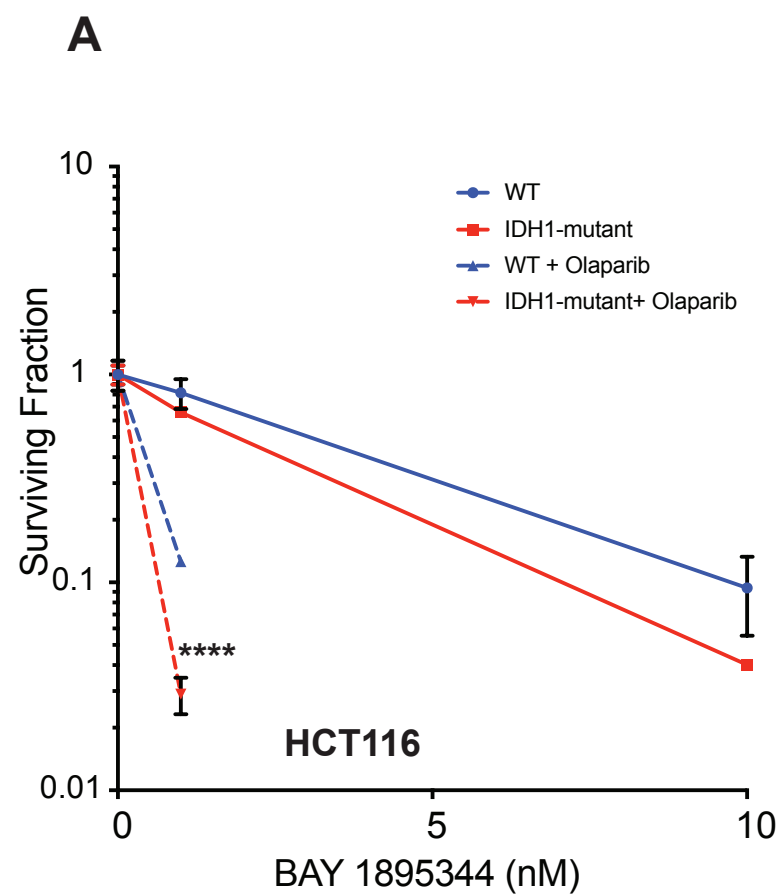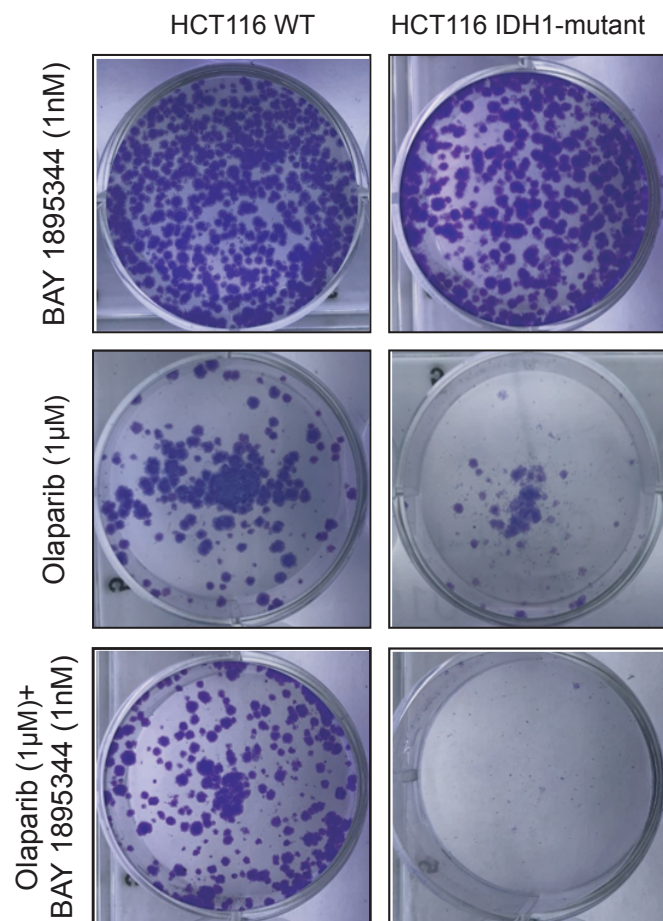

**B**

SW1353 IDH2-mutant

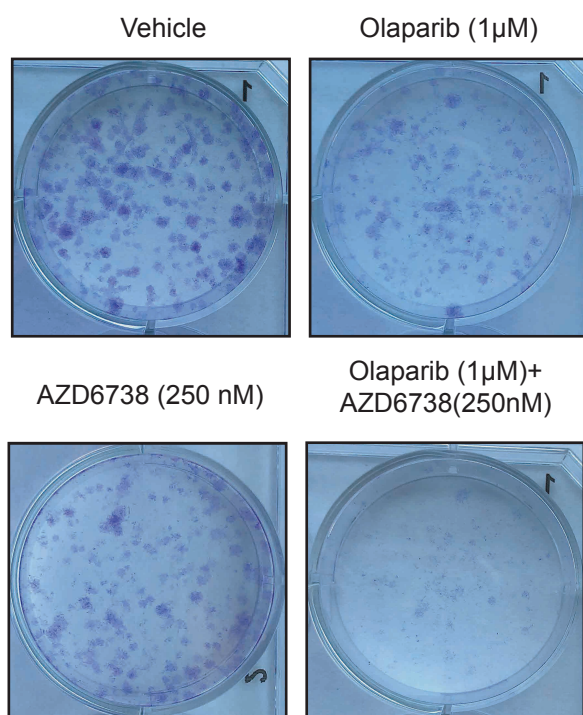

**C**

RBE IDH1-mutant

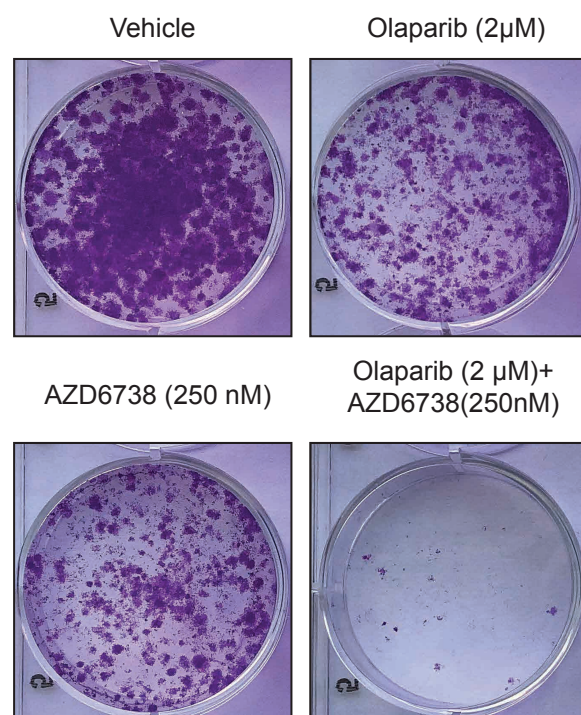

Supplement: zcab018_Supplemental_Files [file zcab018_supplemental_files.zip › S2.pdf]

# Supplemental Figure 3

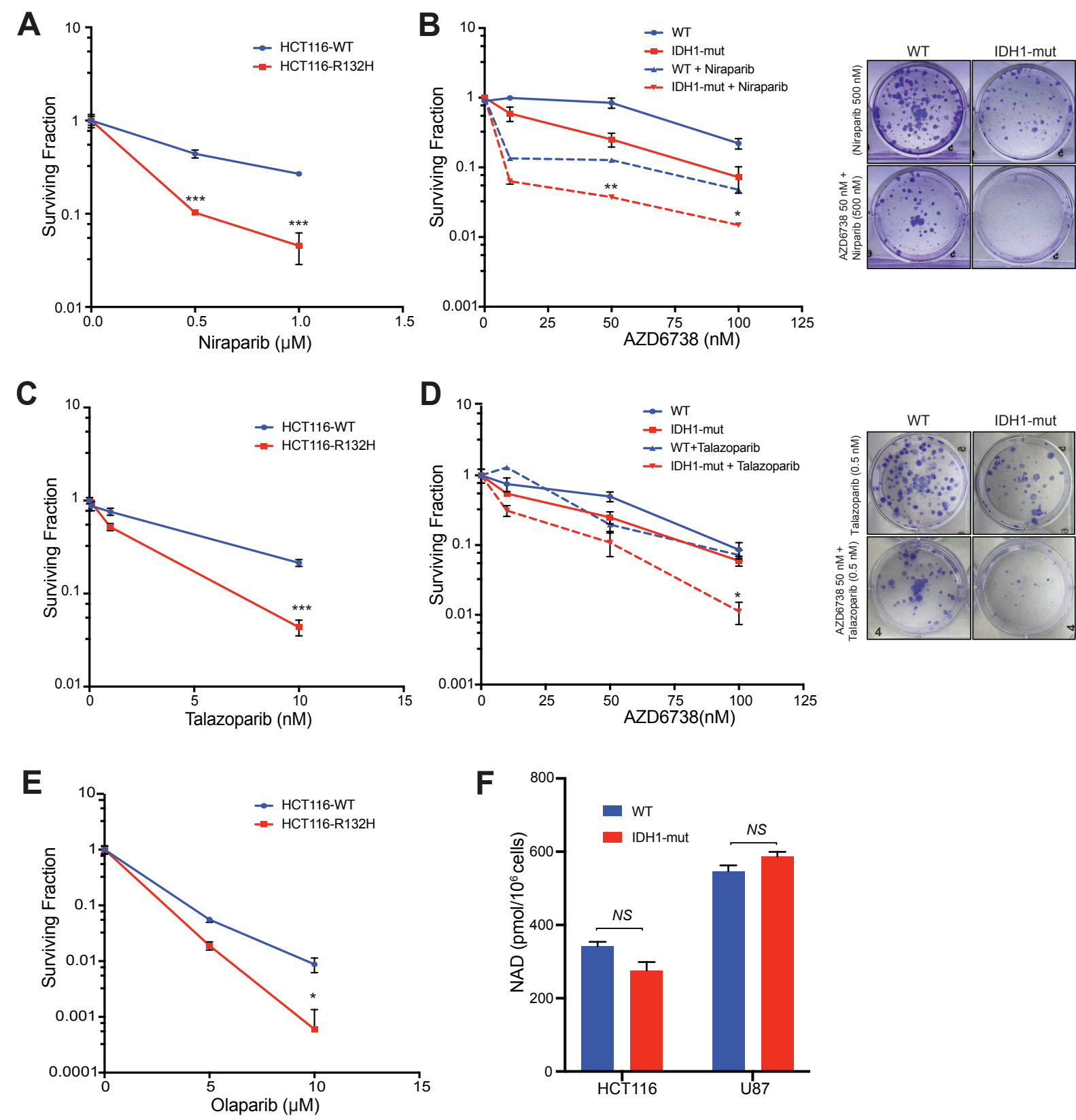

Supplement: zcab018_Supplemental_Files [file zcab018_supplemental_files.zip › S3.pdf]

# Supplemental Figure 4

A

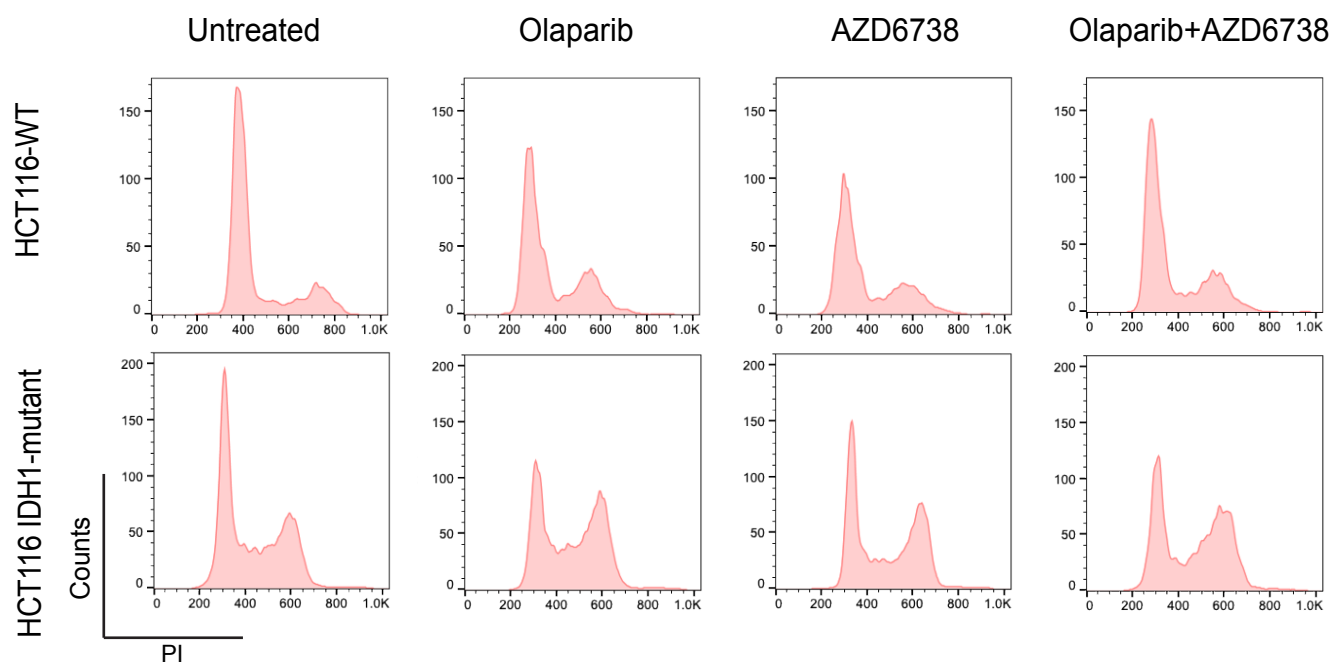

B

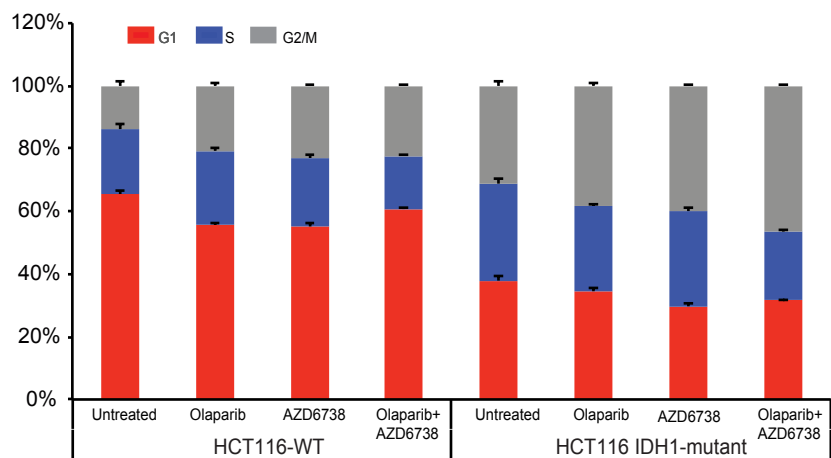

Supplement: zcab018_Supplemental_Files [file zcab018_supplemental_files.zip › S4.pdf]

# Supplemental Figure 5

**A**

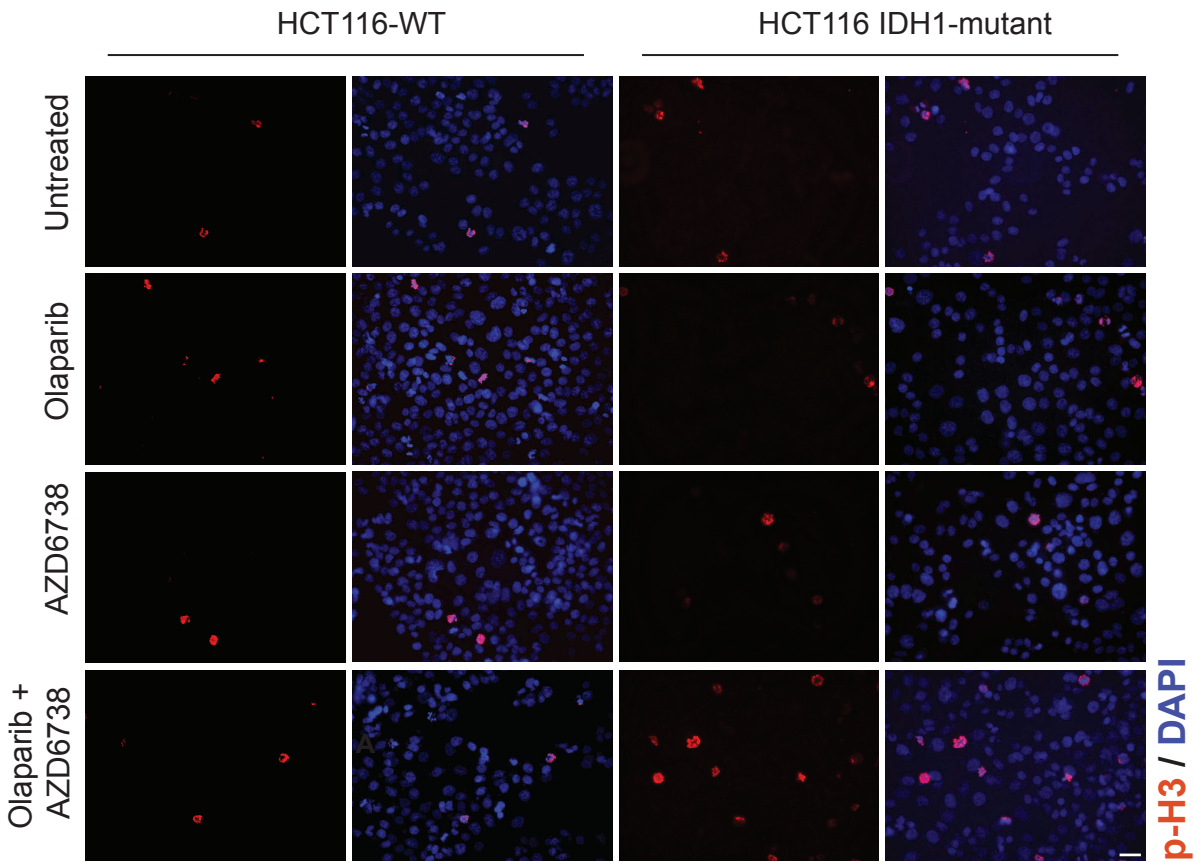

**B**

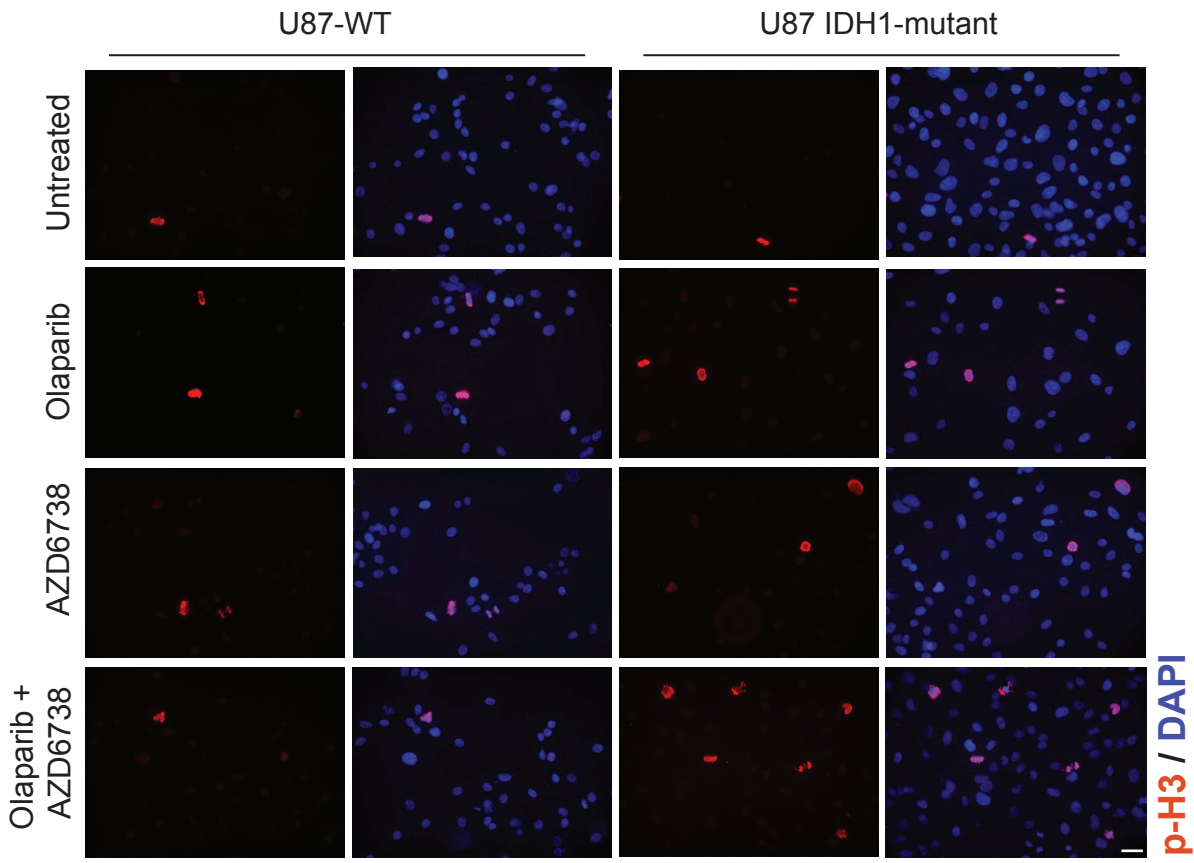

Supplement: zcab018_Supplemental_Files [file zcab018_supplemental_files.zip › S5.pdf]

Supplemental Figure 6

A

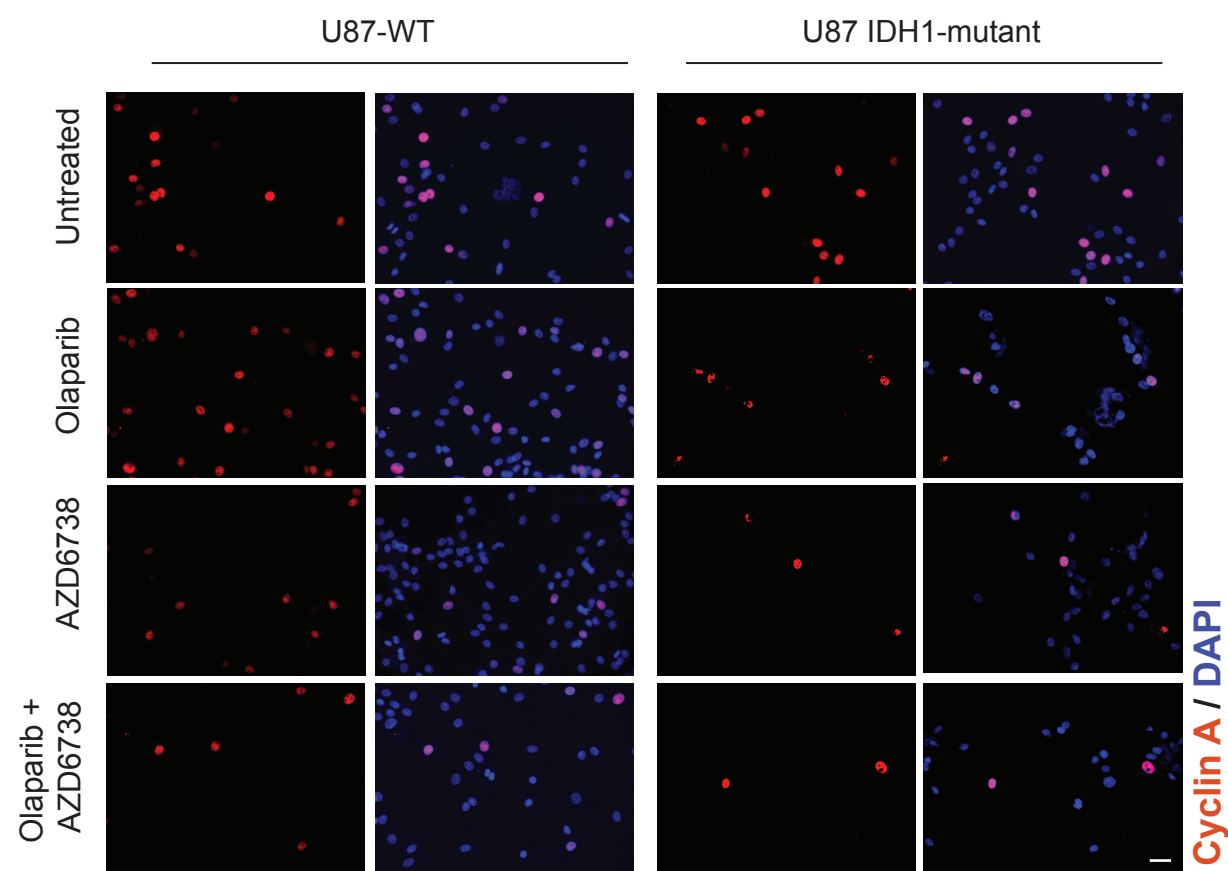

Supplement: zcab018_Supplemental_Files [file zcab018_supplemental_files.zip › S6.pdf]

# Supplemental Figure 7

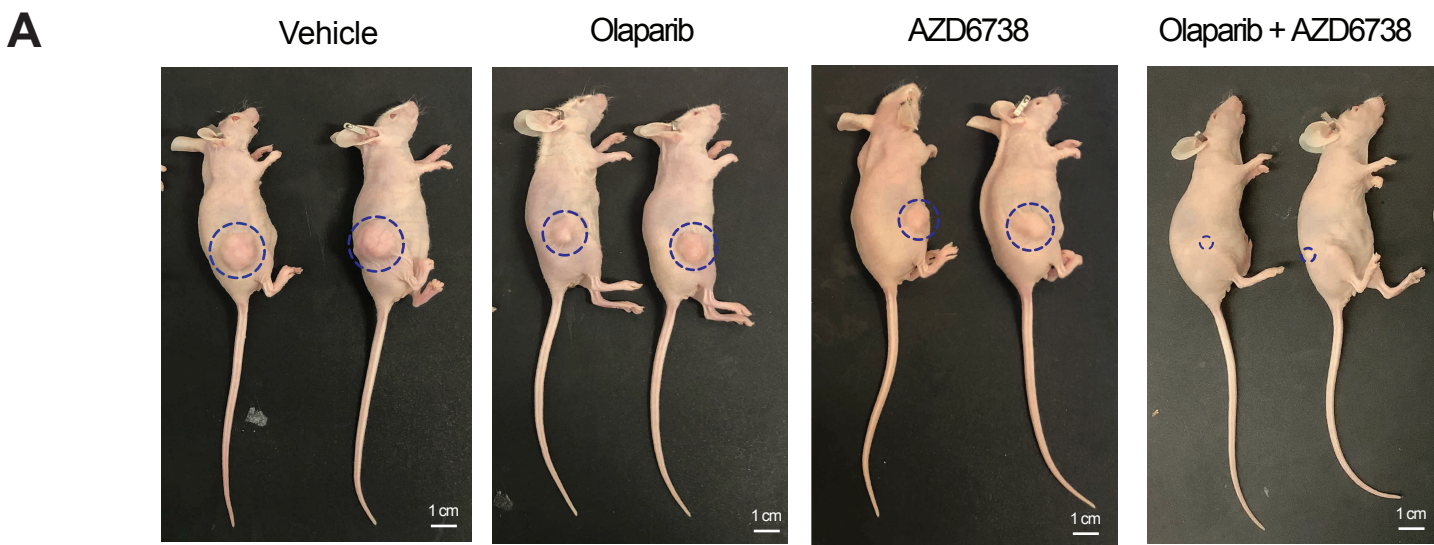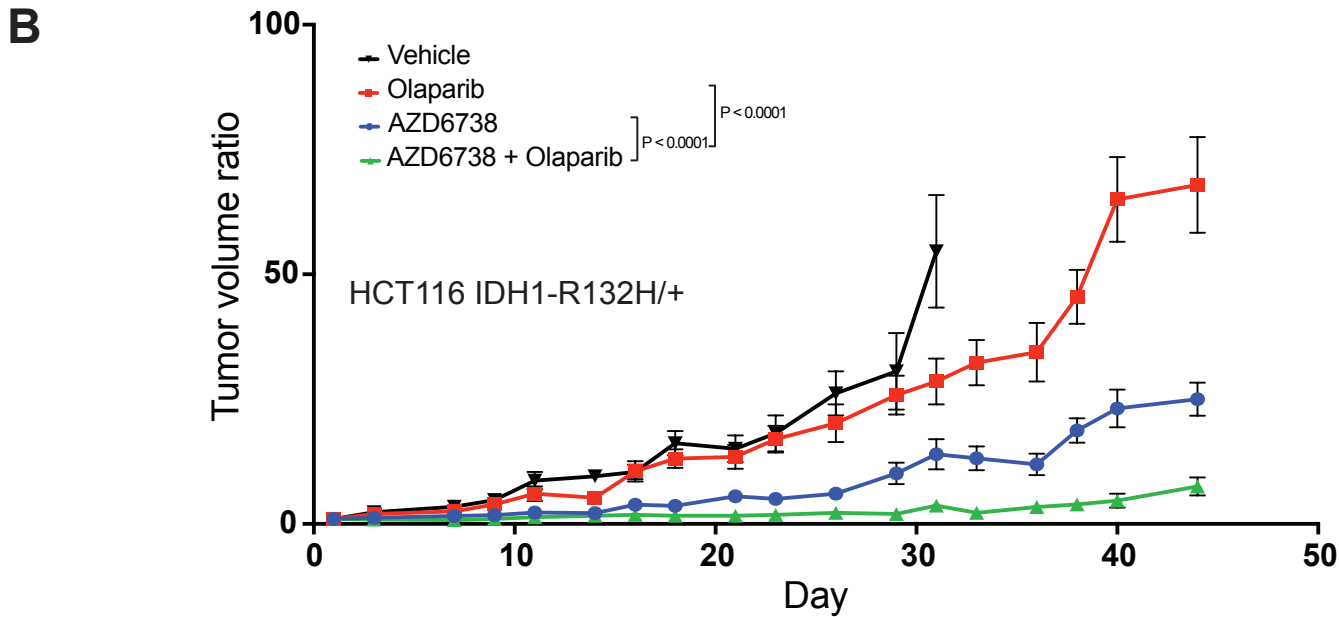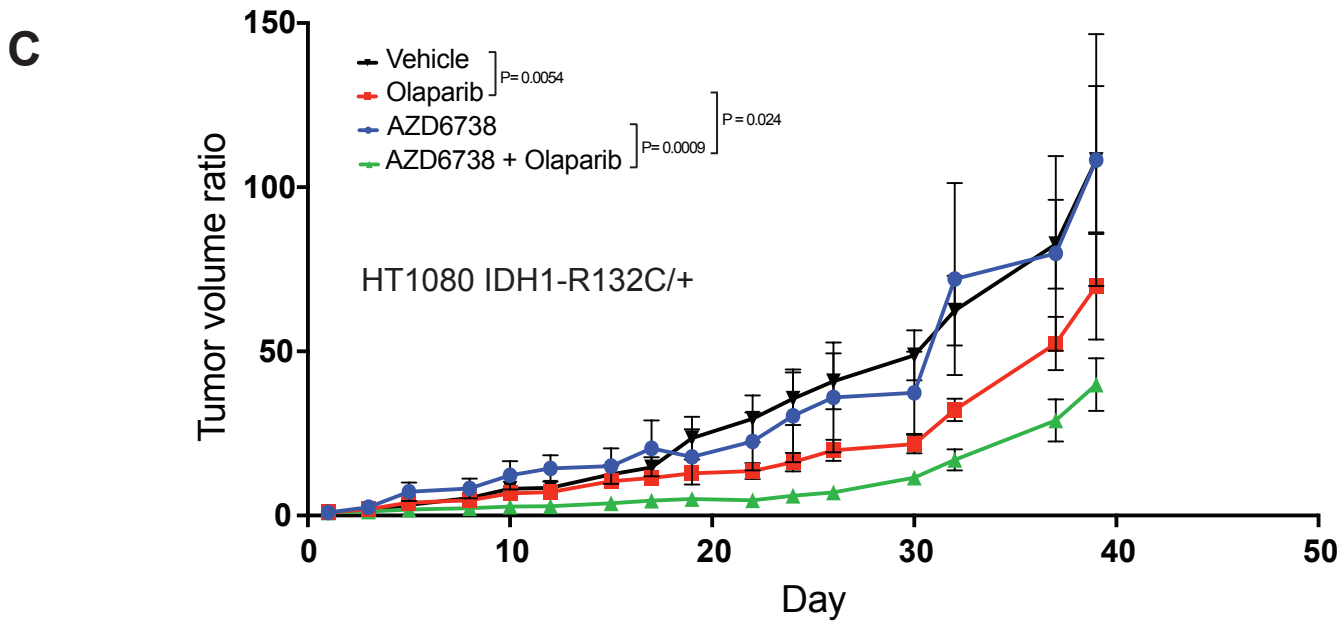

Supplement: zcab018_Supplemental_Files [file zcab018_supplemental_files.zip › S7.pdf]

# Supplemental Figure 8

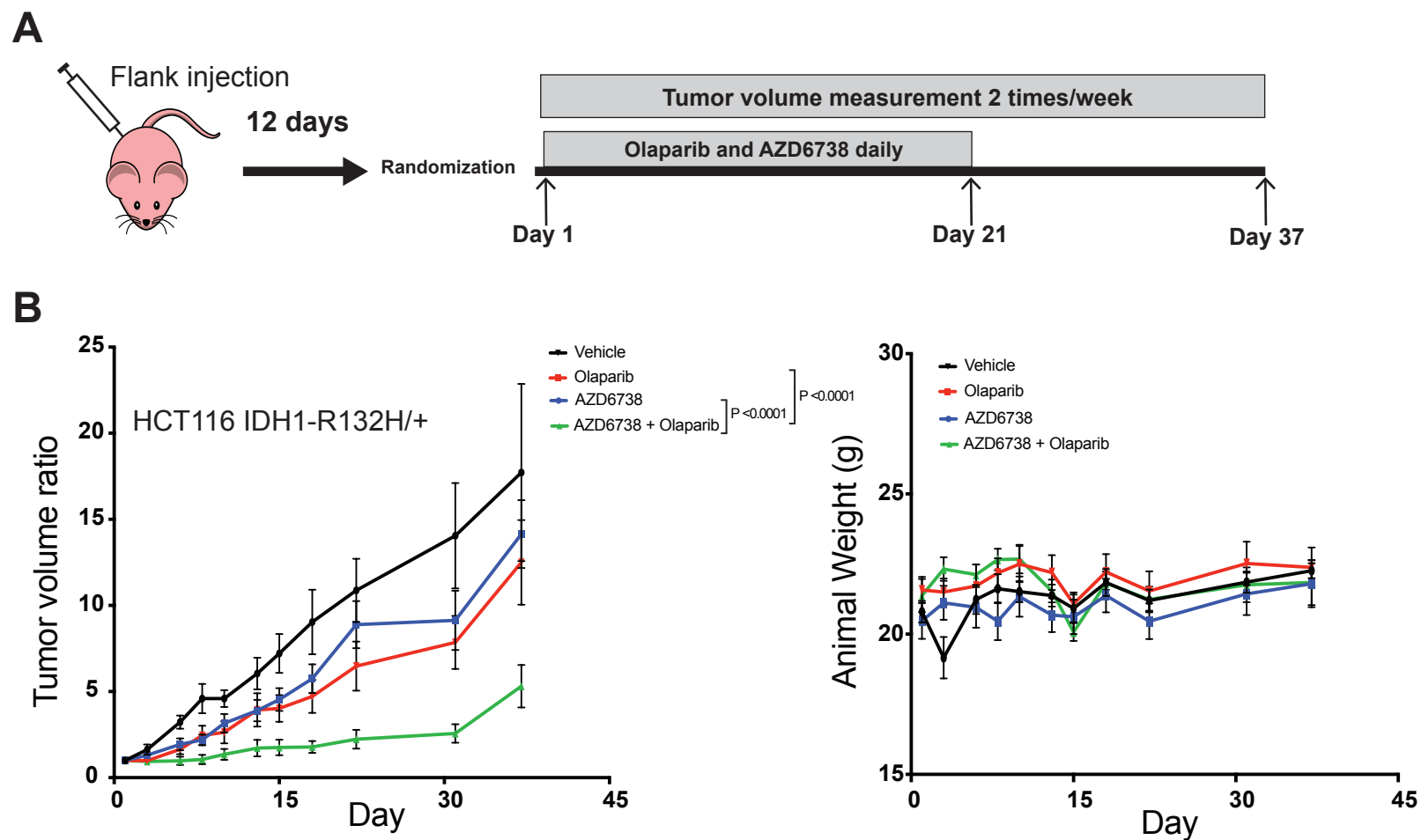

Supplement: zcab018_Supplemental_Files [file zcab018_supplemental_files.zip › S8.pdf]
